# Liver transcriptome analysis in gilthead sea bream upon exposure to low temperature

**DOI:** 10.1186/1471-2164-15-765

**Published:** 2014-09-06

**Authors:** Alba N Mininni, Massimo Milan, Serena Ferraresso, Tommaso Petochi, Patrizia Di Marco, Giovanna Marino, Silvia Livi, Chiara Romualdi, Luca Bargelloni, Tomaso Patarnello

**Affiliations:** Department of Comparative Biomedicine and Food Science, University of Padova, Viale dell’Università 16, 35020 Legnaro, Italy; Department of European and Mediterranean Cultures, Architecture, Environment and Cultural Heritage, University of Basilicata, 75100 Matera, Italy; ISPRA - Italian Institute for Environmental Protection and Research, 00144 Rome, Italy; Department of Biology, University of Padova, via G. Colombo 3, 35131 Padova, Italy

**Keywords:** Gilthead sea bream, Winter syndrome, Cold stress, Microarray, Gene expression, Liver

## Abstract

**Background:**

Water temperature greatly influences the physiology and behaviour of teleost fish as other aquatic organisms. While fish are able to cope with seasonal temperature variations, thermal excursions outside their normal thermal range might exceed their ability to respond leading to severe diseases and death.

Profound differences exist in thermal tolerance across fish species living in the same geographical areas, promoting for investigating the molecular mechanisms involved in susceptibility and resistance to low and high temperatures toward a better understanding of adaptation to environmental challenges. The gilthead sea bream, *Sparus aurata*, is particularly sensitive to cold and the prolonged exposure to low temperatures may lead to the "winter disease", a metabolic disorder that significantly affects the aquaculture productions along the Northern Mediterranean coasts during winter-spring season. While sea bream susceptibility to low temperatures has been extensively investigated, the cascade of molecular events under such stressful condition is not fully elucidated.

**Results:**

In the present study two groups of wild sea bream were exposed for 21 days to two temperature regimes: 16 ± 0.3°C (control group) and 6.8 ± 0.3°C (cold-exposed group) and DNA microarray analysis of liver transcriptome was carried out at different time points during cold exposure.

A large set of genes was found to be differentially expressed upon cold-exposure with increasingly relevant effects being observed after three weeks at low temperature. All major known responses to cold (*i.e.* anti-oxidant response, increased mitochondrial function, membrane compositional changes) were found to be conserved in the gilthead sea bream, while, evidence for a key role of unfolded protein response (UPR) to endoplasmic reticulum (ER) stress, during short- and long-term exposure to cold is reported here for the first time.

**Conclusions:**

Transcriptome data suggest a scenario where oxidative stress, altered lipid metabolism, ATP depletion and protein denaturation converge to induce ER stress. The resulting UPR activation further promotes conditions for cell damage, and the inability to resolve ER stress leads to severe liver dysfunction and potentially to death.

**Electronic supplementary material:**

The online version of this article (doi:10.1186/1471-2164-15-765) contains supplementary material, which is available to authorized users.

## Background

Teleosts are ectotherms and water temperature greatly influences their physiology and behaviour. Within the species-specific thermal tolerance ranges, fishes are able to cope with natural temperature changes, such as daily and seasonal variations. However, close to or beyond the limits of thermal tolerance, physiological adaptation is no longer capable to cope with environmental conditions and fish health and survival are threatened [1, 2]. Thermal stress trigger cellular and organismal responses activating a cascade of events that may lead to pathological conditions and ultimately to death. There are, however, profound differences in thermal tolerance even among fish species with similar geographic distribution [1–3]. Investigating the molecular mechanisms underlying the susceptibility to cold temperatures could provide a better understanding of how organisms either adapt or fail to respond to environmental challenges. The gilthead sea bream, *Sparus aurata* Linneus 1758, although a relatively robust species, is particularly sensitive to cold: when water temperature drops below 12°C, fish become minimally active, reducing food intake and metabolism. For this species the lethal temperature is higher (around 5°C) compared to other teleosts with similar ecological traits and geographic distribution (*e.g.*, European sea bass *Dicentrarchus labrax*, meagre *Argyrosomus regius*) [3, 4].

In the gilthead sea bream, the persistence of low temperatures for a long time may predispose to the "winter disease", a syndrome that involves several factors such as generalized stress, metabolic depression, immune suppression and opportunistic pathogen infections. Along the Northern Mediterranean coasts and lagoons, the outbreak of winter disease may lead to fish mass mortality up to 80% during the winter season, with significant economic losses for semi-intensive and extensive aquaculture production [5–9].

Several aspects of the physiological response to low temperatures in the gilthead sea bream have been investigated both in natural populations and under laboratory conditions [3, 10–13], providing evidence that metabolic depression and oxidative stress occur in different tissues after cold exposure. One of the main pathological consequences of the limited response to cold exposure in the sea bream is the onset of a severe hepatic steatosis, suggesting that liver dysfunction might be at the basis of several winter disease symptoms [10]. However, the cascade of molecular events underlying the response to cold in this temperature-sensitive species is not fully elucidated. Winter disease represents an extreme example of the complex interactions between organismal physiology and environmental stressors, which remain a central issue in aquatic animal ecology [14]. Until recently, biochemical and molecular studies on organismal adaptation to environmental variations were limited to single or few proteins or genes. High-throughput transcriptome analysis, however, now provides an unprecedented view of global transcriptome response to environmental stressors. In particular, DNA microarray and next-generation sequencing, provided detailed molecular information on genomic responses of teleosts to hypoxia [15, 16], heat shock [17–20], cold shock/cold stress [16, 21–28]. In the present study an oligo-DNA microarray representing 19,734 *S. aurata* unique transcripts [29] was used to investigate the response to cold on liver, as this organ exerts important metabolic functions in cold adaptation processes [10, 21]. Fish were exposed for 21 days to 6.8°C in order to simulate environmental conditions that frequently occur during winter in the Northern Mediterranean coastal areas. This critical temperature is near to the lethal threshold reported for this species. The experiment was carried out during winter season, to prevent seasonal impact that might influence the physiological status of the fish. DNA microarray analyses were carried out at four different time points (0 h, 6 h, 24 h and 21 day) in order to compare liver transcriptome profiles in cold-exposed and control individuals (16 ± 0.3°C).

## Results

### Growth and liver condition

The effects of low temperature exposure on growth and liver condition of sea bream are summarized in Table 1. The body weight (BW) was only slightly reduced in cold-exposed groups at the end of the experiment (21d), with no differences observed at starting time (0 h) with controls.

**Table 1:** **Mean value (±SD) of growth parameters and liver condition in control (CTRL) and cold exposed groups (COLD) at the start (0 h) and at the end (21d) of experiment**

Time	0 h		21d	
	CTRL	COLD	CTRL	COLD
**BW (g)**	121.3 ± 17.3^a^	121.2 ± 14.7^a^	118.6 ± 17.5^a^	119.7 ± 20.3^a^
**TL (cm)**	21.2 ± 1.0^a^	21.3 ± 0.9^a^	21.5 ± 1.1^a^	21.3 ± 1.0^a^
**LW (g)**	1.87 ± 0.72^a^	2.07 ± 0.63^a^	1.10 ± 0.45^b^	2.48 ± 0.85^c^
**HSI**	1.52 ± 0.48^a^	1.7 ± 0.44^b^	0.92 ± 0.33^c^	2.04 ± 1.51^d^
**Steatosis (%)**	25^a^	35^a^	5^a^	100^b^

Different letters represent significant differences between groups and sampling time at p<0.05.

Conversely, liver weight and hepatosomatic index (HSI) significantly increased in cold-exposed groups (6.8 ± 0.3°C) at 21d and in comparison with the control groups (16 ± 0.3°C). In most of fish exposed to cold, the liver appeared yellowish and friable and affected by steatosis. No mortality occurred during the trial.

### Microarray analysis

**Figure 1:**
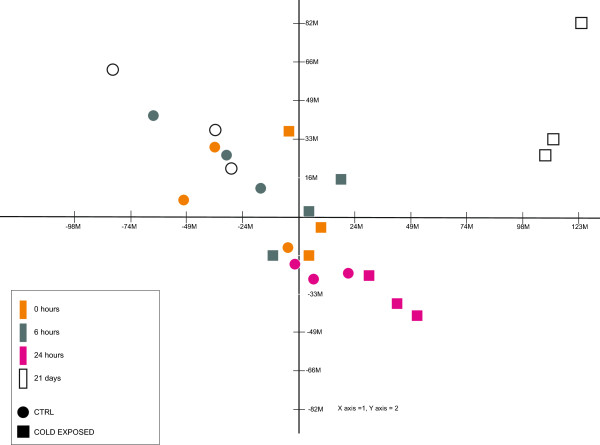
**PCA of** ***S. aurata*** **liver gene expression profiles in controls (circle) and cold groups (square) at the different sampling points.**

A Principal Component Analysis (PCA) of microarray data (Figure 1) showed that 72.4% of the observed transcriptional changes were explained by the first two components and 6.2% by the third component. Since along the first component samples are ordered according to time of exposure, it seems evident already from such an exploratory analysis that long-term exposure to cold (21 days) caused the largest transcriptional variation between cold-exposed and control groups.

**Figure 2:**
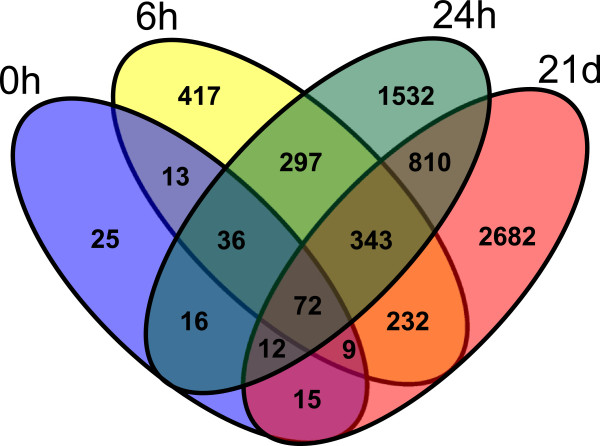
**Venn diagram showing the number of common differentially expressed genes at the four sampling time-points, obtained by SAM.**

To evaluate the effects of low temperature, pair-wise comparisons between cold-exposed and controls at each time point using a two-class SAM test (FDR = 5%; FC > 2; see Methods) were carried out, showing large sets of over- and under-expressed genes in cold-exposed animals, with increasingly relevant effects being observed at later time points (see Table 2). A total of 198 (109 up- and 89 under-expressed), 1,419 (709 over- and 710 under-expressed), 3,124 (1,548 over- and 1,576 under-expressed) and 4,194 (1,835 over- and 2,359 under-expressed) differentially expressed genes (DEGs) were obtained at 0 h, 6 h, 24 h and 21d, respectively (Additional file 1). A substantial overlap was observed between DEGs at different time points (see Figure 2), although just 72 transcripts were significantly differentially expressed along the entire experiment. The response to cold was strong not only in terms of total number of DEGs, but also in the degree of transcriptional changes. Thirty DEGs at 0 h (21 over- and 9 under-expressed), 202 at 6 h (141 over- and 61 under-expressed), 485 at 24 h (240 over- and 245 under-expressed), 660 at 21d (318 over- and 342 under-expressed) showed large FCs (log_2_FC ≥ 2) (see Table 2). The time component in transcriptome regulation appears to be largely due to the effect of cold exposure, as SAM tests performed comparing only control samples across different time points showed no differentially expressed genes (FDR = 5%). Such evidence confirms that the liver transcriptome in control animals is relatively unaffected by the duration of the experiment *per se*, despite the fact that controls were also fasted to increase comparability with cold-exposed animals. A complete list of DEGs is available in Additional file 1.

**Table 2:** **Absolute number of differentially expressed genes (DEGs) at each time-points after temperature drop**

TIME	N° of D.E.G.		Number of D.E.G. at different FC		
			log _2_FC > 3.32	2 ≤ log _2_FC ≤ 3.32	1 ≤ log _2_FC < 2
**0 h**	UP	109	1	20	88
	DOWN	89	0	9	80
**6 h**	UP	709	16	125	568
	DOWN	710	5	56	649
**24 h**	UP	1548	16	224	1308
	DOWN	1576	36	209	1331
**21d**	UP	1835	36	282	1517
	DOWN	2359	34	308	2017

In the right part of the table it is reported the absolute count of genes for each category of log2 fold change (FC) classification.

A useful tool for reducing the complexity of large sets of DEGs is functional annotation and enrichment analysis, which highlights the most significant biological processes across DEGs (Additional file 2). The most significant process at 0 h was GO:0043433 ~ negative regulation of transcription factor activity, with three DEGs: *heme oxygenase (decycling) 1* (HMOX1), *CAAT/enhancer binding protein gamma* (CEBPG), and *DNA-damage-inducible transcript* (DDIT3), more commonly reported as CHOP. All three genes have been reported to be involved in antioxidant response, although DDIT3/CHOP is best known as one of the main effectors of the Unfolded Protein Response (UPR) [30]. Highly relevant to response to oxidative stress, is also *Kelch-like ECH-associated protein 1* (KEAP1, Additional file 1), a cytosolic inhibitor of *nuclear factor erythroid 2-like 2* (NFE2L2, better known as NRF2). Changes in the cellular redox state induce NFE2L2/NRF2 dissociation from KEAP1, its nuclear import and the transcriptional activation of several antioxidant genes, including HMOX1, and of its own inhibitor, KEAP1. Up-regulation of KEAP1 and HMOX1, as observed at 0 h as well as 6 h, 24 h and 21d, suggests an immediate and sustained activation of NFE2L2/NRF2, the main transcription factor involved in the cellular response to oxidative stress.

At 6 h, the most significant enriched Biological Process was GO:0044255 ~ cellular lipid metabolic process represented by 31 DEGs (Additional file 2). Among them, it is listed Glycerol-3-phosphate acyltransferase mitochondrial precursor (GPAT-1), that represents the first step of the metabolic pathway for the synthesis of glycerolipids [31]. Its transcript was significantly over-expressed at 6 h, but also at 24 h and 21d. Similar evidence (over-expression at 6 h, 24 h, and 21d) was observed for *acyl-CoA synthetase long-chain family member 4* (ACSL4), a gene that was reported to be over-expressed in *nonalcoholic fatty liver* (NAFL) [32]. The opposite pattern (constant down-regulation at 6 h-21d) was observed for other genes in the list, *acyl-CoA dehydrogenase C-2 to C-3 short chain* (ACADS), *acyl-CoA dehydrogenase long chain* (ACADL), and *enoyl-coenzyme A hydratase*, all playing important roles in lipid beta-oxidation. On the same line of evidence is the observed under-expression at 6 h-21d of *hydroxysteroid (17-beta) dehydrogenase 4* (HSD17B4), which is involved in the peroxisomal pathway of lipid catabolism. Another gene belonging to the cellular lipid metabolic process is, noteworthy, *peroxisome proliferator-activated receptor delta* (PPARD), which is significantly under-expressed at 6 h and also at 21d. Activation of PPARD was recently reported to attenuate hepatic fat deposition [33].

**Figure 3:**
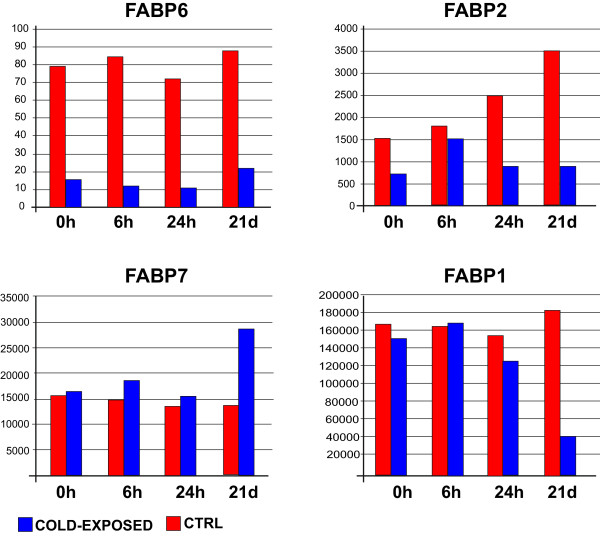
**Average of gene expression values of FABP1, FABP2, FABP6 and FABP7 at each sampling time (0 h = 0 hours; 6 h 6 hours; 24 h 24 hours; 21d = 21 days) in control and cold-exposed groups.**

Finally, a sea bream transcript encoding *fatty acid binding protein 6* (FABP6) was significantly under-expressed at 6 h, as well as at all other time points (Additional file 1). At least three other transcripts encoding FABPs (FABP1, FABP2; FABP7) were found to be under- or over-expressed, especially at 21d (see Figure 3). The most relevant for fatty acid transport within hepatic cells is FABP1, which is the main FABP expressed in the liver [34], as confirmed by absolute fluorescence values in this study (Figure 3). Multiple lines of evidence support the hypothesis that FABP1 acts as a long-chain fatty acid transporter, specifically targeting its ligands to beta-oxidation pathways [34]. In the liver of cold-exposed sea breams FABP1 drops dramatically after 21 days (Figure 3).

In consideration of the extremely large number of differentially expressed genes at 24 h and 21d, enrichment analysis was performed separately for over- and under-expressed DEGs (Additional file 2), although the results were similar when considering all DEGs together (data not shown). At 24 h, the most significantly enriched biological process (BP) for over-expressed genes was GO:0006396 ~ RNA processing with 47 DEGs, the majority of which represented proteins involved in RNA splicing (Additional file 2). In fact, the second most significant BP was GO:0008380 ~ RNA splicing with 34 transcripts. Other noteworthy BPs were GO:0046907 ~ intracellular transport and GO:0008219 ~ cell death. In the latter BP it is included *Growth arrest and DNA damage-inducible beta* (GADD45B) gene, which plays a key role in the response to cellular stress and it has been recently reported to be transcriptionally activated by oxidative stress in hepatic cells [35]. While GADD45B has an essential pro-survival role, other over-expressed genes have opposite actions. For instance, *BCL2-like 14* (BCL2L14) belongs to the pro-apoptotic branch of the Bcl2 family, *cell death-inducing DFFA-like effector c* (CIDEC), *phosphatase and tensin homolog* (PTEN), *tumor protein p53 binding protein 2* (TP53BP2), all favor apoptosis at different levels, while *caspase 6* (CASP6) is one of executioner enzymes for programmed cell death. In the same list is also the transcript encoding CHOP, which was found to be over-expressed as early as 0 h and is among the genes that are differentially expressed at all time points (Additional file 1). In fact, persistent over-expression of CHOP is considered a marker of chronic and/or excessive activation of UPR, and CHOP has been shown to promote cell death [36]. Particularly, the UPR branch leading to CHOP transcription has been shown to be involved in diverse liver diseases (*e.g.* fatty liver disease (FLD) [36, 37]). The most significant BPs for under-expressed genes at 24 h concerned lipid metabolism (GO:0044255 ~ cellular lipid metabolic process, GO:0032787 ~ monocarboxylic acid metabolic process, GO:0006629 ~ lipid metabolic process, GO:0008610 ~ lipid biosynthetic process). Several under-expressed transcripts encoded the same proteins already described for 6 h (ECHDC2, ACSS2, HSD17B4), and additional ones involved in lipid beta- or alpha-oxidation, (*e.g. carnitine O-acetyltransferase*, *enoyl CoA hydratase 1*, *peroxisomal hydroxyacid oxidase 2*), or in the synthesis of phospholipids (*glyceronephosphate O-acyltransferase*, *phosphatidylethanolamine N-methyltransferase*).

Gene expression profiles were even more divergent between controls and cold-exposed fish after 21 days at constant low temperature. While several of BPs enriched at earlier time points remain significant (e.g. lipid metabolism, RNA processing), the most significantly over-represented pathways/processes involve protein metabolism, in particular protein catabolism and folding. Such evidence lends further support to the hypothesis of UPR activation in sea bream hepatic cells under cold stress. In fact, Gene set enrichment analysis (GSEA; see methods) using the reactome "Unfolded Protein Response (UPR)" gene set was marginally significant at 24 h (FDR < 25%) and highly significant at 21d (adjusted p < 0.01) (Additional file 3). In addition to the mentioned CHOP, GSEA identified as significantly over-expressed in cold-exposed fish two other key UPR effectors, *X-box binding protein 1* (XBP1) and *activating transcription factor 4* (ATF4). It has been recently shown that prolonged activation of ATF4 and CHOP leads to cell death through a specific mechanism [38]. While UPR normally decreases protein synthesis, joint over-expression of ATF4 and CHOP activates protein synthesis-related genes, in addition to the UPR transcriptional program. Increased protein synthesis, with consequent ATP depletion, coupled with enhanced oxidative stress, leads to cell death [38]. Remarkably, in cold-exposed animals at 21d nine *aminoacyl-tRNA synthetase* (ARS) genes (YARS, WARS, TARS, AARS, VARS, SARS, GARS, IARS, CARS) are over-expressed, largely overlapping with those reported to be CHOP/ATF4 common targets by Han *et al.* [38]. Likewise, expression of six genes encoding *eukaryotic translation initiation factors* (EIFs) was significantly increased (SAM pairwise test, Additional file 1), including EIF2S2, EIF4G2, and EIF5, which were found to be activated upon CHOP/ATF4 over-expression in human cells.

GSEA also confirmed the main lines of evidence from the analysis of SAM-derived DEGs. When sets comprising genes included in reactomes "Triglyceride Biosynthesis" and "Mitochondrial Fatty Acid Beta Oxidation" were employed, GSEA revealed highly significant enrichment at 21d (Additional file 3). Similar evidence was obtained when analysing the set of putative target genes of the transcription factor CHOP (TFT: V$CHOP_01), while the analogous analysis on genes putatively regulated by the NRF2 (TFT: V$NRF2_01), the key regulator of oxidative stress response, GSEA was highly significant already at 6 h. With regard to proteins involved in protection against reactive oxygen species (ROS), a gene-by-gene analysis revealed that classical anti-oxidant enzymes such as *superoxide dismutase* (SOD1 and SOD2) are not modified at the transcriptional level, while catalase mRNA is significantly under-expressed (Additional file 1). On the other hand, other enzymes with similar function, such as *peroxiredoxin 5* (PRDX5) and *glutathione peroxidase 4* (GPX4) are over-expressed at 21d. Both PRDX5 and GPX4 have a major role in protecting mitochondria from ROS damage.

Metabolic depression and reduced oxygen consumption have been reported in gilthead sea breams exposed to temperatures lower than 12°C [39] and apparently no temperature compensation of metabolic rate occurs in this species. In eurythermal fish such compensation is normally obtained through enhanced aerobic respiration and mitochondrial biogenesis. To explore whether transcripts involved in these processes are differentially expressed in cold-exposed sea breams, GSEA was carried out using two reactome gene sets, "TCA cycle and respiratory electron transport" and "Mitochondrion organization and biogenesis". Genes included in the latter process were marginally enriched at all time points and significantly at 24 h, while transcripts related to aerobic respiration and tricarboxylic acid (TCA) cycle, were differentially expressed at 6 h-21d (Additional file 3). Similarly related to mitochondrial biogenesis are *prohibitins* (PHB1 and PHB2), which have been shown to play a key role in mitochondrial biology [40]. PHB1 was found to be significantly over-expressed under pairwise SAM test in cold-exposed animals at 24 h and 21d.

Another well-known response to cold temperature in fish is the over-expression of *α-* and *β-tubulins* (TUBA and TUBB) and *Δ-9 desaturase* (SCD), which is involved in homeoviscous adaptation (HVA) [21, 28]. In fact, TUBA6 and TUBA1 are over-expressed at all time points (0 h-21d), TUBB5 and TUBB2B at 0 h-21d, while SCD is over-expressed at 24 h and 21d, with a FC > 120 at the latter time point. Likewise, mRNA encoding for *high mobility group box 1* (HMGB1; [21]) was found to be significantly over-expressed at 24 h and 21d (Additional file 1).

### Validation of microarray results using Real-time RT-PCR

Target genes for qRT-PCR analysis were selected considering transcripts with a FC between 0.2 and 13 and represented by two independent probes in the normalized data set. According to these criteria, 10 genes were selected (Additional file 4) and tested on 2 time points.

For all target genes tested, the direction of change in expression was concordant between qRT-PCR data and microarray results. Overall, a statistically significant correlation was obtained comparing the expression levels of each gene across all biological replicates. Five genes showed highly significant correlation coefficients (Spearman rho > 0.8) for both probes (p-value < 0.01) with qPCR data (Additional file 4). One gene (*Glycerol-3-phosfateacyl transferase*; GPAT) exhibited a high correlation (Spearman rho > 0.8, p-value < 0.01) for one probe and a significant correlation (0.7 < rho >0.8 with p-value <0.01) for the other one. One gene (SOD) had a significant correlation for one probe (0.5 < rho < 0.8 with p-value < 0.01) and a positive correlation for the other one (rho = 0.5, p-value > 0.01). The remaining three genes (*26S proteasome complex subunit*, *malate dehydrogenase*, *ACP*) showed a not significant, albeit positive correlation for both probes.

## Discussion

Gene expression analyses of gilthead sea bream exposed to low temperature suggested the conservation of the main mechanisms/processes contributing to setting the thermal limits in ectotherms [41]. These include a broad activation of genes involved in RNA processing, especially at 24 h and 21d. RNA chaperones and, in general, modified RNA processing are thought to be required to compensate for increased stability of RNA secondary structures at low temperatures. Likewise, over-expression of SCD leads to increasing polyunsaturated fatty acids in lipid by-layers, which decrease membrane fluidity contributing to HVA. Up-regulation of HMGB1 was suggested to be involved in compensating the effects of cold on DNA [21], as HMGB1 seems to increase "openness" of DNA to transcription on a genome-wide level. An active transcriptional program for mitochondrial biogenesis was identified as well. Significant alterations in the expression of mRNAs encoding proteins involved in aerobic respiration were found, although, as already observed in other teleosts, the net effect of such changes was not clear.

The investigation of acute stress and mortality at thermal extremes might not reflect the molecular and organismal mechanisms underlying the capacity to respond to less extreme temperatures, which might represent the relevant thermal boundary for a certain species in nature [41]. The experiment presented here builds upon existing knowledge at different levels (biochemical, histological, proteomic) on a well-studied syndrome, the winter disease, which represents a case study for the limited ability to respond to low temperatures. Earlier work already identified tissue- and organ-specific alterations as well as organismal-level effects of low temperatures, suggesting that metabolic unbalances in the liver are one of the central events for cold-induced disease and lethality in the gilthead sea bream [13].

Here, whole-transcriptome evidence is provided on acute and sub-acute reaction in such a key organ, showing a massive response especially after three weeks at low temperature. It should be noted that such a response does not represent the broad alterations of expression profile generally observed in pre-mortal conditions, as no mortality occurred and all fish gradually recovered when water temperature was backed to 16°C [42].

Gene expression analyses highlighted the activation of the anti-oxidant response already at 0 h, and was sustained until 21d (Additional file 2 and Additional file 3).

Recent studies in ectotherms demonstrated increased oxidative stress as temperature moves away from thermal optimum [41, 43]. Noteworthy, the measurement of oxidative stress and the proteomic analyses of sea bream liver exposed to low temperature, revealed a significant increase of lipid peroxidation and nitric oxide in cold-exposed animals. Expression of anti-oxidant proteins was less clear as *glutathione S-transferase* and *catalase* were significantly under-expressed, in agreement with transcriptomic results presented here, while other enzymes potentially involved in oxidative stress response were over-expressed [10]. A vast literature in human medicine suggests a relevant role for oxidative stress in liver disease, especially in hepatic steatosis. Such a knowledge is much pertinent as one of the major histological findings in winter disease is increased lipid content in sea bream liver [11, 44]. More specifically, it has been reported that human hepatocytes exposed to H_2_O_2_ show decreased expression of PPARA and of its target genes [45]. PPARA positively regulates fatty acid oxidation, therefore down-regulation of PPARA and beta-oxidation-related transcripts as observed in human hepatocytes as well as in cold-exposed sea bream liver, might reduce lipid beta-oxidation with consequent lipid accumulation, leading to hepatic steatosis [46, 47]. This hypothesis is consistent with the liver condition of fish observed in this study.

All the mentioned reactions (*i.e.* anti-oxidant response, increased mitochondrial function, stability-compensation at the RNA and DNA level, membrane compositional changes), however, have been already reported in cold-adapted teleosts or in eurythermal fish with good tolerance for low water temperatures. What is different in the gilthead sea bream that makes it unfit for cold? The most interesting finding in the present study is the evidence for a key role of UPR in the acute and sub-acute response to cold. To our knowledge, this is the first time that UPR is observed after exposure to low temperatures. The UPR or endoplasmic reticulum (ER) stress response is a complex mechanism that is conserved across multicellular organisms and is aimed at preventing ER overload with misfolded or damaged proteins [30]. It is particularly important for cells that have high levels of secreted protein synthesis. This is the case of hepatocytes, which actively secrete several serum proteins (*e.g.* albumin, coagulation enzymes, complement, components), and in particular, lipoproteins to re-circulate lipids. UPR signaling consists of three main branches with diverse modes of activation (*e.g.* differential splicing, protein phosphorylation, protein maturation), which converge on a broad transcriptional program to counteract ER stress. Influx of newly synthesized proteins into the ER is decreased, ER-specific chaperones and proteins favoring disulfide bond formation (*e.g*. ERO1-like beta, protein disulfide isomerases) are over-expressed, irreversibly damaged peptides are degraded by up-regulated ERAD components. UPR in hepatocytes has been extensively studied because of its potential relationship with liver disease. ER stress response represents a protective, pro-survival response, which is aimed at restoring cell homeostasis. UPR, however, is a double edge sword. When it is unable to relieve ER overload, chronic UPR activation leads to cell death [36]. Which are the potential triggers for UPR in the case of cold-exposed gilthead sea bream? Low temperature is known to affect protein folding. Protein denaturation at 6°C might not be severe enough to compromise protein function directly, for instance altering enzymatic activity, but it likely represents a stress signal for UPR. Oxidative stress might also contribute to UPR activation as free radicals oxidize cell proteins, and oxidized proteins are unlikely to pass ER quality control. Oxidative protein damage might be paradoxically amplified by some UPR effectors. For instance, ERO1-like proteins (ERO1L alpha and beta) generate ROS in the ER as part of the disulfide bond formation. Remarkably, ERO1L-beta was significantly over-expressed at 21d in cold-exposed fish. Furthermore, protein synthesis and folding require energy. In cold-exposed sea breams ATP might be depleted because of lack of metabolic rate compensation [39]. Finally, prolonged fasting causes depletion of glycogen [12] and in the long term, reduced energy production from glucose could not be compensated by fatty acid oxidation, because such metabolic pathway is down-regulated (PPARA and its target genes). As already mentioned, anti-oxidant response represses beta-oxidation, and UPR appears to act synergistically in this direction through CHOP [48]. Repression of lipid beta-oxidation likely contributes also to accumulation of triglycerides in sea bream hepatocytes. During cold-induced fasting perivisceral lipid stores are mobilized and fatty acids are taken up in the liver [12, 49], this study]. As their catabolic pathway is repressed, fatty acids follow the anabolic route, which is up-regulated at the transcriptional level. The observed up-regulation of PPARG and genes involved in triglyceride biosynthesis (Additional file 1 and GSEA results in Additional file 3) might be a response to increased levels of circulating lipids, although ER stress might contribute as well. The relationships between hepatic lipid biosynthesis and UPR is still controversial, although unresolved ER stress in UPR-effectors null mice is associated with over-expression of genes encoding key enzymes for triglyceride biosynthesis, coupled with significant up-regulation of CHOP and ATF4 [50]. As mentioned earlier, prolonged over-expression of these two transcription factors ultimately leads to cell death through up-regulation of genes involved in protein synthesis, a pattern observed here after three weeks of cold-exposure.

## Conclusions

Low water temperature apparently generates a perfect storm in the gilthead sea bream, where oxidative stress, altered lipid metabolism, ATP depletion, and protein denaturation converge to induce ER stress, UPR mechanisms further promote conditions for cell damage, and the inability to resolve ER stress leads to liver dysfunction and eventually to death.

Under such a scenario, which might be the factors that makes the gilthead sea bream unable to cope with cold, compared to related fish species? Lack of temperature compensation for aerobic metabolism with consequent ATP depletion has been already proposed [39]. Limited capacity to prevent ROS-induced damage in this species might also be hypothesized. Catalase and SODs are key scavenger enzymes, as clearly demonstrated in several studies, yet SODs were unchanged and catalase was down-regulated at the mRNA (this study) and protein level [10]. Finally, UPR might be less effective in the gilthead sea bream, as possibly suggested by similarity in gene expression patterns with UPR-null mice. ER stress response is highly conserved across the animal kingdom, but it also shows significant variation between natural populations in a single species [51]. It is therefore likely that even larger variation occurs between different species.

## Methods

### Animals and experimental conditions

The experiment was performed at the Aquaculture Experimental Centre of Veneto Agricoltura located in Valle Bonello (Rovigo, Italy) from January to March 2008. The experiment was carried out following the standards laid down in the Directive 98/58/EC concerning the protection of animals kept for farming purposes, in accordance with the Commission Recommendation 2007/526/EC on guidelines for the accommodation and care of animals used for experimental and other scientific purposes.

Two groups of wild sea breams (121 g body weight; 21 cm total length) caught in Valle Bonello were kept in duplicate groups in four 5 m^3^ circular recycling tanks and acclimated over one month at 16°C [42]. Fish were then exposed for 21 days to two different temperature treatments: 16 ± 0.3°C (control groups) and 6.8 ± 0.3°C (cold-exposed groups). Because cold-exposed groups stop eating, as naturally occurs during winter season, the control groups were fasted during the trial, in order to distinguish the single effects of low temperature. Warm and cold water temperature conditions were maintained by supplying tanks with two separate re-circulating systems equipped with mechanical and biological filters. Cold water conditions were achieved by inducing two consecutive temperature drops: water was firstly gradually cooled from 16°C to 12°C at a rate of 1°C day ^-1^ and then reduced from 12°C to 6.8°C within 24 h. This temperature was then kept for 21 days. The experimental design is reported in Additional file 5.

### Tissue collection and RNA extraction

Ten fish per treatment were sampled at different intervals during acute (0 hour; 0 h; 6 hours, 6 h; 24 hours, 24 h), and chronic exposure (21 days, 21d) to 6.8°C (see Additional file 5). Fish were firstly anaesthetized in a solution of tricaine methanesulphonate (300 mg/l; MS 222, Finquel) in a 30-litre bucket and then euthanized by severing their spinal cord. Liver samples (about 30 mg) were collected from each specimen, incubated in RNA later at 4°C for 24 h and then stored at -20°C.

Total RNA was extracted from tissue samples using the RNAeasy Mini Kit (Qiagen, Hilden, Germany). RNA concentration was determined using a UV–vis spectrophotometer NanoDrop® ND-1000 (NanoDrop Technologies, Wilmington, USA). RNA integrity and quality was evaluated by means of Agilent 2100 Bioanalyzer (Agilent Technologies, Palo Alto, CA) according to Ferraresso *et al.* [29]. RNA integrity number (RIN) index, that provides a numerical assessment of RNA integrity, was calculated for each sample. This value facilitates the standardization of the quality interpretation; only RNAs with RIN >8 were used for further microarray experiments. Once single RNA samples were extracted and measured, 3 pools (one pool consisting of four individuals, two pools_ of three individuals) per each sampling-time point were prepared. This was aimed at getting a sufficient statistical power which is reached by a minimum of three biological replicates *per* analysed point and at reducing microarray analysis cost.

### Microarray analysis and data validation

To perform DNA microarray analyses the microarray platform GPL6467 has been used [29]. The annotation process of all transcripts represented in microarray was performed as described by Ferraresso et al. (2008). To update the previous annotation, new blastn have been performed against the cDNA database of *Danio rerio*, *Oryzias latipes*, *Tetraodon Nigrovidis*, *Takifugu rubripes* and *Gasterosteus aculeatus* (cut off e-value of <1.0E - 10). A final number of 8,425 out of 19,734 (42.6%) have been annotated in at least one reference database.

Sample labelling and hybridization were performed according to the Agilent One-Color Microarray-Based Gene Expression Analysis protocol for a total of 24 pools (3 biological replicates for each conditions and time point). Briefly, for each pool 500 ng of total RNA were linearly amplified and labeled with Cy3-dCTP. A mixture of 10 different viral poly-adenilated RNAs (Agilent Spike-In Mix) was added to each RNA sample before amplification and labeling, to monitor microarray analysis work-flow. Labeled cRNA was purified with Qiagen RNAeasy Mini Kit, and sample concentration and specific activity (pmol Cy3/μg cRNA) were measured in a NanoDrop® ND-1000 spectrophotometer. A total of 1,650 ng of labeled cRNA was prepared for fragmentation adding 11 μl 10X Blocking Agent and 2.2 μl of 25X Fragmentation Buffer, heated at 60°C for 30 min, and finally diluted by addition with 55 μl 2X GE Hybridization buffer. A volume of 100 μl of hybridization solution was then dispensed in the gasket slide and assembled to the microarray slide (each slide containing four arrays). Slides were incubated for 17 h at 65°C in an Agilent Hybridization Oven, subsequently removed from the hybridization chamber, quickly submerged in GE Wash Buffer 1 to disassembly the slides and then washed in GE Wash Buffer 1 for approximately 1 minute followed by one additional wash in pre-warmed (37°C) GE Wash Buffer 2.

An Agilent G2565BA DNA microarray scanner was used to scan arrays at 5 μm resolution, *Feature Extraction Software 9.5.1* was then used to process and analyse array images. The software returns a series of spot quality measures in order to evaluate the goodness and the reliability of spot intensity estimates. *Spike-in* control intensities (*Spike-In* Viral RNAs) were used to identify the best normalization procedure for each dataset. After cyclic lowess normalization, *spike* intensities are expected to be uniform across the experiments of a given dataset. Filtering and normalization analyses were performed using R statistical software [52]. Gene expression data were deposited in the GEO database under accession numbers GSE51442.

Principal Component Analysis (PCA) was performed considering the entire gene expression dataset using TMeV software. SAM statistical tests [53] were carried out to identify differentially expressed genes between cold and control groups (False Discovery Rate (FDR) equal to 5% and fold-change (FC) threshold of log_2_ FC =1, which corresponds to FC = 2). A more systematic functional interpretation of differentially transcribed genes was obtained through an enrichment analysis using Database for Annotation, Visualization, and Integrated Discovery (DAVID) software [54, 55] considering the GO Biological Process Database. In order to obtain IDs compatible with DAVID, BLASTN (cut off e-value of 1.0 E-5) was used on ENSEMBL database to search for significant matches with the transcriptomes of the 5 teleost species (see Additional file 6). For annotating sea bream transcripts showing significant matches with more than one species, priority was assigned first to *G. aculeatus* (GA), then *T. rubripes* (TR), *T. nigroviridis* (TN), *O. latipes* (OL) and *D. rerio* (DR). Retrieved protein sequences from Ensembl genome browser ( http://www.ensembl.org/biomart/martview), were then employed to carry out a Blastp (cut off e-value of 1.0 E-3) between teleost homologues of sea bream transcripts and human Ensembl protein database. Finally, Ensembl Human Gene IDs were obtained from the corresponding Ensembl protein entries using the BIOMART data mining tool ( http://www.ensembl.org/biomart/martview/).

Homologous genes were found for 7,295 transcripts in at least one teleost species, while human homologous have been identified for 6,835 transcripts. These identifiers were used to define a "gene list" and a "background" in the bioinformatic tool DAVID, corresponding to differentially transcribed genes and to all the transcripts that were represented on the array, respectively. Annotation steps performed for DAVID functional analyses are summarized in Additional file 6. The functional annotation was obtained for genes differentially expressed between cold and control groups setting DAVID for gene count = 2 and ease = 0.1. Gene set enrichment analysis (GSEA) was performed on the entire gene expression dataset using the Gene Set Enrichment Analysis v2.0.13 software [56] downloaded from the Broad Institute [57]. GSEA analysis was performed by using gene symbols retrieved by blastx against UniProt database. For enrichment analysis REACTOME gene sets were downloaded from the C2 collections in MsigDB v3.1 (Molecular Signature Database). Pathway analysis was performed for each gene set independently. Signal2Noise metric was employed for gene ranking, and 1000 permutations were applied for pathway p-value assignment.

In order to validate microarray data, ten target genes (see Additional file 7) were selected for real-time RT-PCR analysis. qPCR assays were performed in a total of 12 samples (control and cold-exposed groups at 24 h and 21d), the same employed for microarray experiments. For each selected target gene and for the reference gene (RPL13a, Ribosomal Protein L13a), a qRT-PCR assay was designed using the SYBRGreen system of detection. Gene-specific primers were defined for each transcript with the Primer3 software. To design intron-spanning primers, putative intron-exon boundaries were inferred by comparison with homologs of sea bream genes present in high-quality draft genome sequences from other fish species (*Tetraodon nigroviridis, Danio rerio, Oryzias latipes, Takifugu rubripes* and *Gasterosteus aculeatus*).

One microgram of total RNA for each sample was reverse transcribed to cDNA using Superscript II (Invitrogen™). An aliquot (2.5 μl) of diluted (1:40) cDNA template was amplified in a final volume of 10 μl, containing 5 μl of mastermix SYBRGreen® Master 2X (Invitrogen) and 0.25 μl of each gene-specific primer (10 μM). The amplification protocol consisted of an initial step of 2 min at 50°C and 10 min at 95°C, followed by 45 cycles of 10 s at 95°C and 30 s at 60°C. All experiments were carried out in a LightCycler® 480 (Roche Diagnostics). To evaluate the efficiency of each assay, standard curves were constructed amplifying two-fold serial dilutions of the same cDNA which was used as calibrator (Sa_CAL). For each sample, the Cp (Crossing point) was used to determine the relative amount of target gene; each measurement was made in duplicate, and normalized to the reference gene (RPL13a), which was also measured in duplicate. RPL13a was chosen as reference gene in qRT-PCR assays as it is considered a housekeeping gene, and it did not exhibit any significant change in microarray data between exposed to low temperature and control individuals. To validate pool homogeneity and exclude particular differences between single samples that constitute the same pool, these were individually tested in real-time RT-PCR. In addition, to make sure that all targets genes were expressed in the calibrator, Sa_CAL, the internal control for each amplification reaction, was constructed pooling the same amount of three RNA liver samples for each time point and condition.

A non parametric Spearman rank-correlation test was applied for each target gene on all 12 samples employed for microarray experiments in order to assess the correlation between the expression values measured respectively with real-time RT-PCR and microarray probes. Statistical analyses were performed using SPSS ver. 12.0.

### Post-mortem examination

Post-mortem examination was performed on fish experimentally exposed to low temperature and controls to evaluate growth and liver steatosis (fatty liver). Body weight (g) and total length (cm) were recorded at the beginning and end of trial.

The severity of steatosis was evaluated based on liver appearance, colour and consistency [58]. Liver was further weighed and hepatosomatic index (HSI) calculated as HSI = LW/BW*100, where LW is the liver weight (g) and BW is the body weight.

The Student’s t-test was applied to determine the effects of cold exposure on growth, LW and HSI and the χ2-test to test the frequency of liver steatosis

### Data accessibility

Gene expression analyses were performed using the Agilent-016251 *Sparus aurata* oligo microarray (GEO accession: GPL6467). Microarray raw and normalized fluorescence values were deposited in the GEO database ( http://www.ncbi.nlm.nih.gov/geo) under accession number GSE51442.

## Electronic supplementary material

Additional file 1: **Differentially expressed genes found between control and cold-exposed gilthead seabream at 0 h, 6 h, 24 h and 21 d.** Probes reported in red were over-expressed in cold-exposed samples. Probes reported in green were under-expressed in cold-exposed samples. Fold change, q-value (%)are also reported. For each significant probes has been reported the annotation considering amino-acid non redundant database (cut off e-value of <1.0E - 10), and Ensembl protein database of *Danio rerio*, *Oryzias latipes*, *Tetraodon Nigrovidis*, *Takifugu rubripes* and *Gasterosteus aculeatus*. (XLSX 1009 KB)

Additional file 2: **Enriched Biological Processes (BP) among differentially expressed genes at 0 h, 6 h, 24 h and 21 d.** At 24 h and 21d, enrichment analysis was carried separately for over- and under-expressed DEGs. (XLSX 54 KB)

Additional file 3: **Gene set enrichment analysis (GSEA) of DEGs found between control and cold-exposed gilthead seabream at 0 h, 6 h, 24 h and 21 d.** (XLS 36 KB)

Additional file 4: **Table A.** Correlation between microarray and real-time RT-PCR expression data, using Spearman rank-correlation test. **Table B.** Comparison of gene expression values between qRT-PCR and microarray probes for selected target genes. (PDF 263 KB)

Additional file 5: **Experimental design representing fish acclimatization and sampling times.** (PDF 1 MB)

Additional file 6: **Information about the annotation of gilthead seabream transcriptome performed in order to obtain IDs compatible for the functional analyses.** Blastn (cut off e-value of <1.0E - 10) have been performed against the cDNA database of *Danio rerio (DR)*, *Oryzias latipes (OL)*, *Tetraodon Nigrovidis (TN)*, *Takifugu rubripes* (TR) and *Gasterosteus aculeatus* (GA). Retrieved protein sequences from Ensembl genome browser, Blastp was then carried out (cut off e-value of 1.0 E-3) between teleost homologues of sea bream transcripts and human Ensembl protein database. (PDF 170 KB)

Additional file 7: **Primer sequences of the ten transcripts analysed by real-time RT-PCR to validate Microarray data.** 26S: *26S proteasome complex subunit*; FAS: *Fatty acid synthase*; GPAT: *Glycerol-3-phosfateacyl transferase*; PKC: *Protein kinase C*; KAT: *3-ketoacylCoA thiolase*; ILF2: *Interleukin-2*; MDH: *Malate dehydrogenase*; SOD: *Superoxide dismutase*; CD59; ACP; RPL13a: *Ribosomal Protein L13a.* (PDF 84 KB)

## Abbreviations

HSI: Hepatosomatic index
FC: Fold change
FDR: False Discovery rate
DEGs: Differentially expressed genes
HMOX1: Heme oxygenase (decycling) 1
CEBPG: CAAT/enhancer binding protein gamma
DDIT3/CHOP: DNA-damage-inducible transcript
UPR: Unfolded Protein Response. KEAP1, Kelch-like ECH-associated protein 1
NFE2L2/NRF2: Nuclear factor erythroid 2-like 2
GPAT-1: Glycerol-3-phosphate acyltransferase mitochondrial precursor
ACSL4: Acyl-CoA synthetase long-chain family member 4
NAFL: Nonalcoholic fatty liver
ACADS: Acyl-CoA dehydrogenase C-2 to C-3 short chain
ACADL: Acyl-CoA dehydrogenase long chain
HSD17B4: Hydroxysteroid (17-beta) dehydrogenase 4
PPARD: Peroxisome proliferator-activated receptor delta
PPARG: Peroxisome proliferator-activated receptor gamma
PPARA: Peroxisome proliferator-activated receptor alpha
FABP6: Fatty acid binding protein 6
FABP1: Fatty acid binding protein 1
FABP2: Fatty acid binding protein 2
FABP7: Fatty acid binding protein 7
BP: Biological process
GADD45B: Growth arrest and DNA damage-inducible beta
BCL2L14: BCL2-like 14
CIDEC: Cell death-inducing DFFA-like effector c
PTEN: Phosphatase and tensin homolog
TP53BP2: Tumor protein p53 binding protein 2
CASP6: Caspase 6
FLD: Fatty liver disease
XBP1: X-box binding protein 1
ATF4: Activating transcription factor 4
IRE1: Inositol-requiring enzyme 1
ERAD: Endoplasmic reticulum associated degradation
ARS: Aminoacyl-tRNA synthetase
EIF: Eukaryotic translation initiation factors
ROS: Reactive oxygen species. SOD1, Superoxide dismutase 1
SOD2: Superoxide dismutase 2
GSEA: Gene set enrichment analysis
PRDX5: Peroxiredoxin 5
GPX4: Glutathione peroxidase 4
PHB1: Prohibitins 1
TUBA: α- tubulins
TUBB: β-tubulins
SCD: Δ-9 desaturase
HVA: Homeoviscous adaptation
GPAT: Glycerol-3-phosfateacyl transferase
26S: 26S proteasome complex subunit
MDH: Malate dehydrogenase
ACP: Acyl-carrier-protein
HMGB1: High mobility group box 1.
