# Supplementary material for: Liver transcriptome analysis in gilthead sea bream upon exposure to low temperature
Source: BMC Genomics. 2014 Sep 6;15(1):765. doi: 10.1186/1471-2164-15-765 (PMC4167152; doi:10.1186/1471-2164-15-765)
Supplement: Supplementary file 4 — Additional file 4: Table A. Correlation between microarray and real-time RT-PCR expression data, using Spearman rank-correlation test. Table B. Comparison of gene expression values between qRT-PCR and microarray probes for selected target genes. (PDF 263 KB) [file 12864_2014_6443_MOESM4_ESM.pdf]

TABLE A. Correlation between microarray and real-time RT-PCR expression data

SAPD ID	Gene Name	Spearman's rho<br>Probe_1/Probe_2	qPCR/Probe_1	qPCR/Probe_2
**SAPD23647**	26S proteasome complex subunit (26S)	0.930**	0.347	0.270
**SAPD21212**	Fatty acid synthase (FAS)	0.951**	0.895**	0.951**
**SAPD10252**	Glycerol-3-phosfateacyl transferase GPAT)	0.804**	0.937**	0.747**
**SAPD23278**	Protein kinase C (PKC)	0.893**	0.930**	0.858**
**SAPD20246**	3-ketoacylCoA thiolase (KAT)	0.944**	0.867**	0.832**
**SAPD11592**	ACP	0.958**	0.298	0.326
**SAPD20810**	CD-59	0.965**	0.839**	0.881**
**SAPD18820**	Interleukin-2 (ILF2)	0.973**	0.879**	0.821**
**SAPD02236**	Malate dehydrogenase (MDH)	0.818**	0.053	0.249
**SAPD22939**	Superoxide dismutase (SOD)	0.453	0.502	0.712**

** $p < 0.01$

TABLE B. Comparison of gene expression values between qRT-PCR and microarray probes for selected target genes.

		SAPD23647				SAPD21212				SAPD10252				SAPD23278				SAPD20246			
		24h		21d		24h		21d		24h		21d		24h		21d		24h		21d	
		Cold	Ctrl	Cold	Ctrl	Cold	Ctrl	Cold	Ctrl	Cold	Ctrl	Cold	Ctrl	Cold	Ctrl	Cold	Ctrl	Cold	Ctrl	Cold	Ctrl
Probe 2	Mean expression	0.758	0.839	2.440	1.004	2.369	1.067	5.137	0.732	6.451	1.220	5.555	0.990	0.428	1.072	2.593	1.076	0.312	1.294	0.412	0.903
	SD	0.089	0.142	0.378	0.031	0.230	0.094	0.364	0.325	1.089	0.217	0.137	0.036	0.153	0.068	0.912	0.410	0.038	0.380	0.070	0.141
Probe 1	Mean expression	0.645	0.849	2.495	1.029	1.777	0.953	6.306	0.775	5.361	0.983	7.513	0.941	0.282	0.964	3.119	1.864	0.231	1.128	0.376	0.972
	SD	0.100	0.133	0.098	0.060	0.323	0.173	0.573	0.314	0.521	0.050	0.362	0.222	0.082	0.080	1.216	1.139	0.025	0.120	0.002	0.159
qPCR	Mean expression	1.374	1.240	4.084	0.839	2.261	0.938	9.112	0.725	3.849	0.932	7.094	0.833	0.307	0.800	5.882	2.263	0.212	1.536	0.564	0.731
	SD	0.343	0.208	1.780	0.154	0.153	0.182	3.983	0.249	0.492	0.097	2.491	0.215	0.090	0.262	1.231	1.468	0.062	0.873	0.163	0.245

		SAPD11592				SAPD20810				SAPD18820				SAPD02236				SAPD22939			
		24h		21d		24h		21d		24h		21d		24h		21d		24h		21d	
		Cold	Ctrl	Cold	Ctrl	Cold	Ctrl	Cold	Ctrl	Cold	Ctrl	Cold	Ctrl	Cold	Ctrl	Cold	Ctrl	Cold	Ctrl	Cold	Ctrl
Probe 2	Mean expression	0.858	0.924	1.779	0.961	0.259	0.885	0.568	0.888	2.244	0.768	9.313	0.860	0.430	1.036	0.609	1.190	1.334	1.029	1.654	0.999
	SD	0.180	0.099	0.109	0.033	0.031	0.141	0.048	0.415	0.530	0.372	0.902	0.302	0.125	0.090	0.143	0.167	0.128	0.177	0.221	0.081
Probe 1	Mean expression	0.926	0.941	1.640	0.917	0.257	0.920	0.578	0.944	1.990	0.935	11.86	0.858	0.526	1.105	0.618	1.068	0.971	0.901	1.491	1.104
	SD	0.131	0.061	0.199	0.095	0.046	0.072	0.056	0.415	0.236	0.092	0.550	0.501	0.192	0.092	0.125	0.080	0.052	0.091	0.204	0.206
qPCR	Mean expression	1.352	1.191	2.754	1.166	0.414	0.923	0.707	0.730	2.566	0.948	15.34	0.897	0.555	0.746	0.711	0.967	1.652	1.013	2.469	0.924
	SD	0.158	0.176	0.027	0.304	0.252	0.112	0.197	0.374	0.344	0.056	1.564	0.121	0.249	0.226	0.384	0.033	0.182	0.179	0.572	0.269
