# Supplementary figures and images for: Liver transcriptome analysis in gilthead sea bream upon exposure to low temperature
Source: BMC Genomics. 2014 Sep 6;15(1):765. doi: 10.1186/1471-2164-15-765 (PMC4167152; doi:10.1186/1471-2164-15-765)

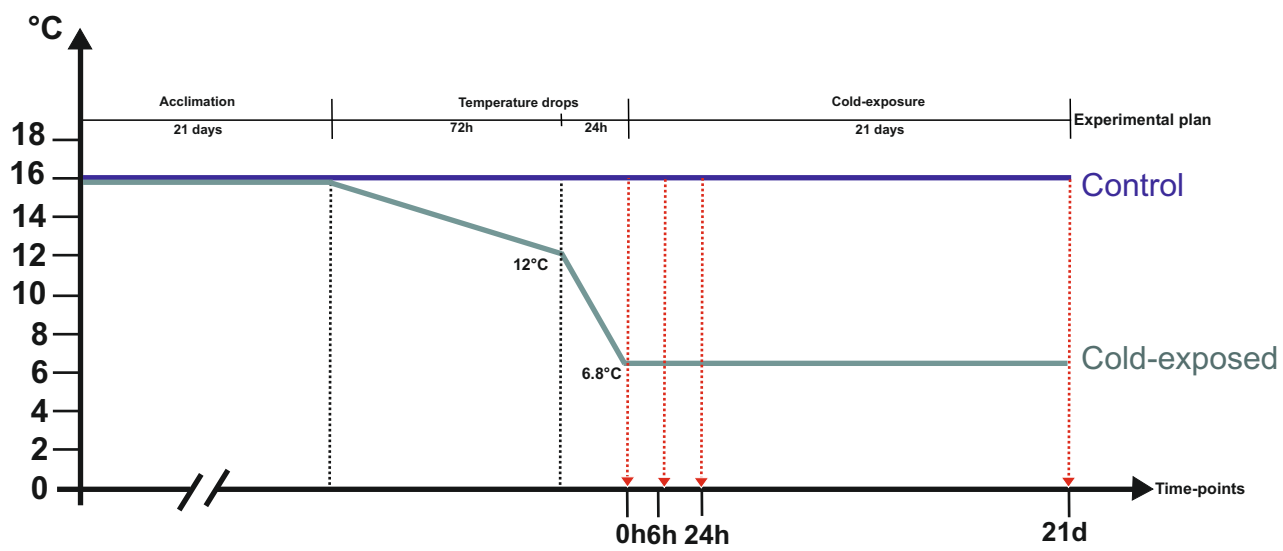

Supplement: Supplementary file 5 — Additional file 5: Experimental design representing fish acclimatization and sampling times. (PDF 1 MB) [file 12864_2014_6443_MOESM5_ESM.pdf]
