# Supplementary material for: Liver transcriptome analysis in gilthead sea bream upon exposure to low temperature
Source: BMC Genomics. 2014 Sep 6;15(1):765. doi: 10.1186/1471-2164-15-765 (PMC4167152; doi:10.1186/1471-2164-15-765)
Supplement: Supplementary file 6 — Additional file 6: Information about the annotation of gilthead seabream transcriptome performed in order to obtain IDs compatible for the functional analyses. Blastn (cut off e-value of <1.0E - 10) have been performed against the cDNA database of Danio rerio (DR), Oryzias latipes (OL), Tetraodon Nigrovidis (TN), Takifugu rubripes (TR) and Gasterosteus aculeatus (GA). Retrieved protein sequences from Ensembl genome browser, Blastp was then carried out (cut off e-value of 1.0 E-3) between teleost homologues of sea bream transcripts and human Ensembl protein database. (PDF 170 KB) [file 12864_2014_6443_MOESM6_ESM.pdf]

**STATISTICAL**	**GA**	**OL**	**TN**	**DR**	**TR**
N° of annotated SAPD (blastn; e-value of 1.0 E-5)	6,186	5,338	5,274	3,986	5,491
N° of SAPD annotated with at least one species	7,295				
N° of SAPD with human homologs gene	6,835				
