# Supplementary material for: Liver transcriptome analysis in gilthead sea bream upon exposure to low temperature
Source: BMC Genomics. 2014 Sep 6;15(1):765. doi: 10.1186/1471-2164-15-765 (PMC4167152; doi:10.1186/1471-2164-15-765)
Supplement: Supplementary file 7 — Additional file 7: Primer sequences of the ten transcripts analysed by real-time RT-PCR to validate Microarray data. 26S: 26S proteasome complex subunit; FAS: Fatty acid synthase; GPAT: Glycerol-3-phosfateacyl transferase; PKC: Protein kinase C; KAT: 3-ketoacylCoA thiolase; ILF2: Interleukin-2; MDH: Malate dehydrogenase; SOD: Superoxide dismutase; CD59; ACP; RPL13a: Ribosomal Protein L13a. (PDF 84 KB) [file 12864_2014_6443_MOESM7_ESM.pdf]

Gene name	Primer sequence
KAT	For_CCAGCAATCACaGAAGCTCTTA
	Rev_CTTTGCcACaGCCAGgTACT
GAPT	For_GATCCAgTAcGGAGTTCTCTACG
	Rev_AAAGGGGCTCgGGaAACT
MDH	For_CCGTCTGGACCACAACAGA
	Rev_GCCCCAGATGATCACATTCT
26S	For_CAACTGGGATGATGACAAtG
	Rev_cGTcAGCCGtTAtGAtGTCTC
FAS	For_TGTGTGCCTTCaTCCAGCA
	Rev_AGTGCAGtaAGTCtCGGCTGAT
PKC	For_AACAGGTCGGCAAGTTCAAG
	Rev_CAGATCCCGaTACACGATGC
ILF2	For_CaTACAAGAAGGGCACCATGA
	Rev_TGGACAGCACTTCaGTAGGG
CD59	For_gCAGGAGTGCACGTATGAAG
	Rev_TGGGtGAGGCGaGAGTTATC
SOD	For_ACtCTCAgTGGgCccttgT
	Rev_CGTTGCCcGTCTTTAGACTC
ACP	For_TTGGACCAGGTGGAGATCAT
	Rev_AaACGTCCTTCTTGTCTGCaA
RPL13a	For_TTCTACCGCAACAAGCTGAA
	Rev_CCCTGACAGTCCTCCAGAAG
